# Lack of Association of Two Common Polymorphisms rs2910164 and rs11614913 with Susceptibility to Hepatocellular Carcinoma: A Meta-Analysis

**DOI:** 10.1371/journal.pone.0040039

**Published:** 2012-06-29

**Authors:** Zhongxia Wang, Yin Cao, Chunping Jiang, Guang Yang, Junhua Wu, Yitao Ding

**Affiliations:** 1 Department of Hepatobiliary Surgery, Affiliated Drum Tower Hospital of Nanjing University Medical School, Nanjing, Jiangsu Province, China; 2 Jiangsu Province’s Key Medical Center for Hepatobiliary Surgery, Nanjing, Jiangsu Province, China; 3 School of Medicine, Nanjing University, Nanjing, Jiangsu Province, China; University of North Carolina School of Medicine, United States of America

## Abstract

**Background:**

Single nucleotide polymorphisms (SNPs) in microRNA-coding genes may participate in the process of carcinogenesis by altering the expression of tumor-related microRNAs. It has been suggested that two common SNPs rs2910164 in miR-146a and rs11614913 in miR-196a2 are associated with susceptibility to hepatocellular carcinoma (HCC). However, published results are inconsistent and inconclusive. In the present study, we performed a meta-analysis to systematically summarize the possible association between the two SNPs and the risk for HCC.

**Methodology/Principal Findings:**

We conducted a search of case-control studies on the associations of SNPs rs2910164 and/or rs11614913 with susceptibility to HCC in PubMed, EMBASE, ISI Web of Science, Cochrane Central Register of Controlled Trials, ScienceDirect, Wiley Online Library and Chinese National Knowledge Infrastructure databases. Data from eligible studies were extracted for meta-analysis. HCC risk associated with the two polymorphisms was estimated by pooled odds ratios (ORs) and 95% confidence intervals (95% CIs). 5 studies on rs2910164 and 4 studies on rs11614913 were included in our meta-analysis. Our results showed that neither allele frequency nor genotype distribution of the two polymorphisms was associated with risk for HCC in all genetic models. Similarly, subgroup analysis in Chinese population showed no association between the two SNPs and the susceptibility to HCC.

**Conclusions/Significance:**

This meta-analysis suggests that two common SNPs rs2910164 and rs11614913 are not associated with the risk of HCC. Well-designed studies with larger sample size and more ethnic groups are required to further validate the results.

## Introduction

Primary liver cancer is the sixth most common cancer worldwide (748,300 new cases per year). Due to its poor prognosis and high fatality rates, the incidence and mortality rates are almost equal. Primary liver cancer causes 695,900 deaths every year, making it the third most common cause of cancer-related deaths [1]. Hepatocellular carcinoma (HCC), which represents the dominant histological type, accounts for 70–85% of primary malignancies in liver [2]. Epidemiological survey suggests that East and South-East Asia and Middle and Western Africa have the highest prevalence of HCC with almost half of the new cases and deaths in China [3]. Although chronic infection of hepatitis B virus and hepatitis C virus are considered as the major risk factors of HCC, the etiology of HCC is still yet to be clarified [4]. Current studies indicated genetic factors may also contribute to the etiology of HCC [5].

MicroRNAs (miRNAs) are small non-coding, single-stranded RNA molecules with typical length of ∼22 nucleotides. Functioning as post-transcriptional regulators, miRNAs complementarily bind to target mRNAs and negatively regulate their stability and transcriptional efficiency [6]. Numerous studies have demonstrated that alteration of miRNAs may play an important role in the pathogenesis of HCC [7]. Genetic alterations of miRNAs may have distinguished significance in HCC initiation and progression since one single miRNA may have hundreds of gene targets. Even a slight variation in the function or expression of a miRNA may affect a wide spectrum of mRNA targets including many oncogenes and tumor suppressor genes [8].

Single nucleotide polymorphisms (SNPs) in miRNA-coding genes may have effects on either the expression or the function of miRNAs by altering the secondary structure of miRNA precursors, consequently leading to the aberrant expression of a series of target genes and contributing to cancer susceptibility [9]. Studies on the associations between SNPs in miRNAs and human cancer have provided new insights into the molecular mechanisms of cancer development. To date, several groups have reported polymorphisms rs2910164 in miR-146a and rs11614913 in miR-196a2 could be the biomarkers of susceptibility to HCC [10]–[17]. However, the associations observed between miR-146a rs2910164 and miR-196a2 rs11614913 polymorphisms and the risk for HCC are controversial and inconclusive. Since the relatively small sample size of a single study may not have enough power to detect slight effects of these SNPs on HCC, meta-analysis may provide more credible evidence by systematically summarizing existed data. Although several meta-analyses have reported associations between the two common SNPs and susceptibility to various cancers [18]–[25], the clinical heterogeneity between the included studies on cancers from diverse histological natures may limit the reliability of the conclusions. Moreover, these meta-analyses did not include all of eligible studies on HCC, thus may limit the efficacy of detecting potential associations between the two SNPs and HCC risk. In the present study, we conducted a meta-analysis in order to derive more precise and comprehensive estimation of the associations between the SNPs miR-146a rs2910164 and miR-196a2 rs11614913 and susceptibility to HCC.

## Methods

### Searching

We carried out a publication search in PubMed, EMBASE, ISI Web of Science, Cochrane Central Register of Controlled Trials, ScienceDirect, Wiley Online Library and Chinese National Knowledge Infrastructure (CNKI) databases with the following search terms: (miR-146a OR miR-196a2 OR rs2910164 OR rs11614913) AND (hepatocellular carcinoma OR liver cancer OR hepatoma), by two independent investigators (Zhongxia Wang and Yin Cao, last search update: May 14, 2012). Publication date and publication language were not restricted in our search. Reference lists were examined manually to further identify potentially relevant studies. We contacted the corresponding authors of conference abstracts without sufficient data for additional information by e-mail. If no reply or the author refused to provide the data required in this meta-analysis, the study was excluded. All studies matching the inclusion criteria were retrieved for further examination and data extraction. Investigators include experts in hepatobiliary surgery, biologists and qualified graduate researchers. All of the investigators have received training in literature search, statistics and evidence-based medicine.

### Selection

Studies included in our meta-analysis had to meet all the following criteria: (1)evaluated the associations between the SNPs miR-146a rs2910164 and/or miR-196a2 rs11614913 and susceptibility to HCC, (2) studied on human beings, (3)in a case-control design, (4)detailed genotype data were provided for the calculation of odds ratio (OR) and 95% confidence interval (95% CI), (5)if serial studies of the same population from the same group were reported, the latest study was included. We assessed the methodological qualities of included studies by the description of study population, detailed genotyping methods, the set of controls and cases and related statistical methods. Included studies had to meet the following criteria regarding methodology: (1) participants recruited from comparable populations with similar demographic background in control and case groups, (2) proper diagnostic criteria to determine the disease status, (3) Hardy-Weinberg equilibrium (HWE) reached in control population, (4) proper method to determine the genotypes with detailed description of method used, (5) appropriate statistical analysis performed, (6) detailed allele and genotype frequencies reported.

### Data Abstraction

Two investigators (Zhongxia Wang and Yin Cao) independently extracted data from the included studies. Data extracted from eligible studies included the first author’s name, publication date, country origin, ethnicity, genotyping method, total numbers of cases and controls and genotype frequencies of cases and controls. The two investigators checked the data extraction results and reached consensus on all of the data extracted. If different results were generated, they would check the data and have a discussion to come to an agreement. Three senior investigators (Yitao Ding, Chunping Jiang and Guang Yang) were invited to the discussion if disagreement still existed.

### Quantitative Data Synthesis

For each study, HWE was evaluated using the goodness-of-fit chi-square test. *P*<0.05 was considered representative of a departure from HWE. ORs and 95% CIs were calculated to assess the strength of the association between miR-146a rs2910164 or miR-196a2 rs11614913 polymorphism and susceptibility to HCC. Pooled ORs were calculated for allele frequency comparison (miR-146a rs2910164: C versus G, miR-196a2 rs11614913: C versus T), additive model (miR-146a rs2910164: GC versus GG, CC versus GG, and miR-196a2 rs11614913: TC versus TT, CC versus TT), dominant model (miR-146a rs2910164: GC/CC versus GG, and miR-196a2 rs11614913: TC/CC versus TT), and recessive model (miR-146a rs2910164: CC versus GC/GG, and miR-196a2 rs11614913: CC versus TC/TT), respectively. The significance of pooled ORs was determined by *Z*-test and *P*<0.05 was considered as statistically significant. Statistical heterogeneity among the studies was checked by chi-square-based *Q*-test. A *P-*value greater than 0.10 for *Q-*test indicates no significant heterogeneity existed among studies [26], so that the pooled OR was estimated by the fixed-effects model (the Mantel-Haenszel method) [27]; otherwise, if the heterogeneity was significant, the random-effects model (the DerSimonian and Laird method) was employed [28]. Sensitivity analysis was carried out by deleting one single study each time to examine the influence of individual data set on the pooled ORs. Publication bias of literatures was assessed using funnel plots and Egger’s test. An asymmetric plot suggests a possible publication bias and the *P* value of Egger’s test less than 0.05 was considered representative of statistically significant publication bias [29]. All of the statistical tests were performed with STATA software version 11.0 (STATA Corporation, College Station, TX, USA).

## Results

### Study Characteristics

**Figure 1:**
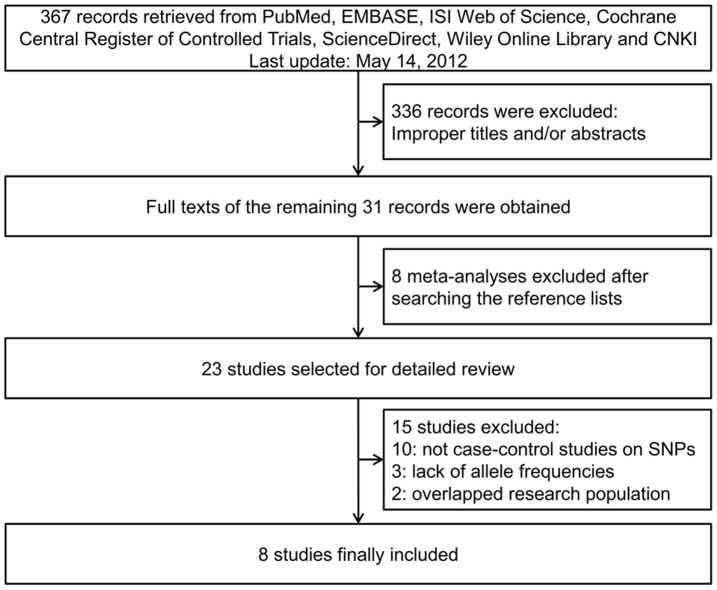
Flow diagram of study identification.

A total of 367 articles were retrieved after first search in PubMed, EMBASE, ISI Web of Science, Cochrane Central Register of Controlled Trials, ScienceDirect, Wiley Online Library and CNKI databases. As shown in Figure 1, after our selection, 8 case-control studies fulfilled the inclusion criteria [10]–[17]. The qualities of the studies were considered acceptable for our meta-analysis. Characteristics of included studies are summarized in Table 1. In the study from Zhang *et al*. [17], genotype frequencies of miR-146a rs2910164 and miR-196a2 rs11614913 were presented separately, so that data of each SNP were extracted separately for meta-analysis. Therefore, a total of 5 studies [10]–[13], [17] involving 1,912 cases and 2,149 controls for miR-146a rs2910164 and 4 studies [14]–[17] involving 1,790 cases and 1,635 controls for miR-196a2 rs11614913 were ultimately analyzed in our meta-analysis. For miR-146a rs2910164, 4 studies [11]–[13], [17] were carried out in Asian population (Chinese population) while 1 study [10] was from Turkish population. As for miR-196a2 rs11614913, there were 3 studies [15]–[17] on Asians (Chinese population) and 1 study [14]_ENREF_14 on Turkish population. Several genotyping methods were employed in the studies including polymerase chain reaction-restriction fragment length polymorphism (PCR-RFLP), polymerase chain reaction-ligation detection reaction (PCR-LDR) and polymerase chain reaction-primer introduced restriction analysis (PCR-PIRA). The genotypes distribution in the controls was in agreement with HWE in all of the included studies. MOOSE checklist was generated to provide detailed description of this meta-analysis (Table S1).

**Table 1:** Characteristics of studies included in the meta-analysis.

Author	Year	Country	Ethnicity	SNP	Genotypingmethods	No.(cases/controls)	HWE†	rs2910164/rs11614913Case(%)			rs2910164/rs11614913Control(%)		
								GG/TT	GC/TC	CC/CC	GG/TT	GC/TC	CC/CC
Akkiz	2011	Turkey	Caucasian	rs2910164	PCR-RFLP	222/222	0.384	137(61.7)	75(33.8)	10(4.5)	144(64.9)	67(30.1)	11(5.0)
Xiang	2012	China	Asian	rs2910164	PCR-RFLP	100/100	0.506	27(27.0)	45(45.0)	28(28.0)	21(21.0)	46(46.0)	33(33.0)
Xu	2008	China	Asian	rs2910164	PCR-RFLP	479/504	0.119	80(16.7)	241(50.3)	158(33.0)	58(11.5)	249(48.4)	197(39.1)
Zhou	2011	China	Asian	rs2910164	PCR-RFLP	186/483	0.056	33(17.7)	86(46.2)	67(36.0)	71(14.7)	254(52.6)	158(32.7)
Akkiz	2011	Turkey	Caucasian	rs11614913	PCR-RFLP	185/185	0.492	22(11.9)	86(46.5)	77(41.6)	40(21.6)	87(47.0)	58(31.4)
Li	2010	China	Asian	rs11614913	PCR-RFLP	310/222	0.402	82(26.4)	150(48.4)	78(25.2)	78(35.1)	102(46.0)	42(18.9)
Qi	2010	China	Asian	rs11614913	PCR-LDR	361/391	0.869	100(27.7)	179(49.6)	82(22.7)	102(26.1)	197(50.4)	92(23.5)
Zhang	2011	China	Asian	rs2910164	PCR-PIRA	925/840	0.149	156(16.9)	450(48.6)	319(34.5)	151(18.0)	386(45.9)	303(36.1)
				rs11614913	PCR-PIRA	934/837	0.972	277(29.6)	449(48.1)	208(22.3)	239(28.6)	417(49.8)	181(21.6)

† Hardy-Weinberg equilibrium (HWE) was evaluated using the goodness-of-fit chi-square test. *P* values were presented. *P*<0.05 was considered representative of a departure from HWE. SNP, single nucleotide polymorphism.

### Meta-analysis of the Association between miR-146a rs2910164 Polymorphism and Susceptibility to HCC

The association between miR-146a rs2910164 polymorphism and susceptibility to HCC was analyzed in five independent studies with 1,912 cases and 2,149 controls. Results of the meta-analysis are shown in Table 2. *Q*-test in all of the genetic models showed no significant heterogeneity. Therefore, the pooled ORs were calculated using fixed-effects model. No significant association between miR-146a rs2910164 polymorphism and susceptibility to HCC was identified in any of the genetic models (C versus G: OR = 0.932, 95% CI 0.851–1.022, *P* = 0.135; GC versus GG: OR = 0.954, 95% CI 0.804–1.133, *P* = 0.592; CC versus GG: OR = 0.850, 95% CI 0.701–1.031, *P* = 0.098; GC/CC versus GG: OR = 0.925, 95% CI 0.787–1.087, *P* = 0.344; CC versus GC/GG: OR = 0.904, 95% CI 0.788–1.037, *P* = 0.150).

**Table 2:** Summary of pooled ORs in the meta-analysis of SNP rs2910164.

Genetic Model	Population	Pooled OR [95% CI] *P*	*P_h_* †
C versus G	Overall	0.932 [0.851–1.022] 0.135	0.212
	Chinese	0.920 [0.836–1.012] 0.088	0.178
GC versus GG	Overall	0.954 [0.804–1.133] 0.592	0.152
	Chinese	0.911 [0.754–1.101] 0.336	0.142
CC versus GG	Overall	0.850 [0.701–1.031] 0.098	0.218
	Chinese	0.845 [0.693–1.029] 0.095	0.128
Dominant	Overall	0.925 [0.787–1.087] 0.344	0.119
GC/CC versus GG	Chinese	0.883 [0.739–1.056] 0.173	0.116
Recessive	Overall	0.904 [0.788–1.037] 0.150	0.450
CC versus GC/GG	Chinese	0.904 [0.786–1.039] 0.155	0.297

† *P_h_*, *P-*value for heterogeneity test. OR, odds ratio; CI, confidence interval.

Four out of the five included studies were conducted in Chinese population. In ethnicity subgroup analysis, no association was found to be statistical significant in Chinese population in any genetic model (C versus G: OR = 0.920, 95% CI 0.836–1.012, *P* = 0.088; GC versus GG: OR = 0.911, 95% CI 0.754–1.101, *P* = 0.336; CC versus GG: OR = 0.845, 95% CI 0.693–1.029, *P* = 0.095; GC/CC versus GG: OR = 0.883, 95% CI 0.739–1.056, *P* = 0.173; CC versus GC/GG: OR = 0.904, 95% CI 0.786–1.039, *P* = 0.155) (Table 2).

### Meta-analysis of the Association between miR-196a2 rs11614913 Polymorphism and Susceptibility to HCC

4 studies involving 1,790 cases and 1,635 controls were assessed for the association between miR-196a2 rs11614913 polymorphism and HCC risk. Significant statistical heterogeneity was identified in the comparison of allele frequency, TC versus TT, CC versus TT and in the dominant model so that random-effects model was used in these models. Fixed-effects model was used in the recessive model. However, for rs11614913, none of the genetic models produced significant association between rs11614913 polymorphism and HCC risk (C versus T: OR = 1.149, 95% CI 0.937–1.409, *P* = 0.182; TC versus TT: OR = 1.121, 95% CI 0.855–1.470, *P* = 0.408; CC versus TT: OR = 1.318, 95% CI 0.881–1.974, *P* = 0.180; TC/CC versus TT: OR = 1.200, 95% CI 0.874–1.647, *P* = 0.260; CC versus TC/TT: OR = 1.132, 95% CI 0.965–1.326, *P* = 0.127). The results are summarized in Table 3.

**Table 3:** Summary of polled Ors in the meta-analysis of SNP rs11614913.

Genetic Model	Population	Pooled OR [95% CI] *P*	*P_h_* †
C versus T	Overall	1.149 [0.937–1.409] 0.182‡	0.010
	Chinese	1.064 [0.886–1.278] 0.508‡	0.061
TC versus TT	Overall	1.121 [0.855–1.470] 0.408‡	0.079
	Chinese	0.998 [0.844–1.179] 0.978	0.188
CC versus TT	Overall	1.318 [0.881–1.974] 0.180‡	0.012
	Chinese	1.124 [0.796–1.588] 0.507‡	0.081
Dominant	Overall	1.200 [0.874–1.647] 0.260‡	0.015
TC/CC versus TT	Chinese	1.065 [0.812–1.396] 0.651‡	0.080
Recessive	Overall	1.132 [0.965–1.326] 0.127	0.175
CC versus TC/TT	Chinese	1.074 [0.905–1.275] 0.413	0.301

† *P_h_*, *P-*value for heterogeneity test.

‡ Random-effects model was used when the *p*-value for heterogeneity test ≤0.10, otherwise the fixed-effect model was used. OR, odds ratio; CI, confidence interval.

Similarly, subgroup analysis in Chinese population showed no significant association between SNP rs11614913 and susceptibility to HCC in Chinese population (C versus T: OR = 1.064, 95% CI 0.886–1.278, *P* = 0.508; TC versus TT: OR = 0.998, 95% CI 0.844–1.179, *P* = 0.978; CC versus TT: OR = 1.124, 95% CI 0.796–1.588, *P* = 0.507; TC/CC versus TT: OR = 1.065, 95% CI 0.812–1.396, *P* = 0.651; CC versus TC/TT: OR = 1.074, 95% CI 0.905–1.275, *P* = 0.413)(Table 3).

### Publication Bias

Funnel plot and Egger’s test were performed to assess the publication bias of the literature. Symmetrical funnel plots were obtained in both of the SNPs tested in all of the models. Egger’s test further confirmed the absence of publication bias in this meta-analysis (*P*>0.05).

### Sensitivity Analysis

**Figure 2:**
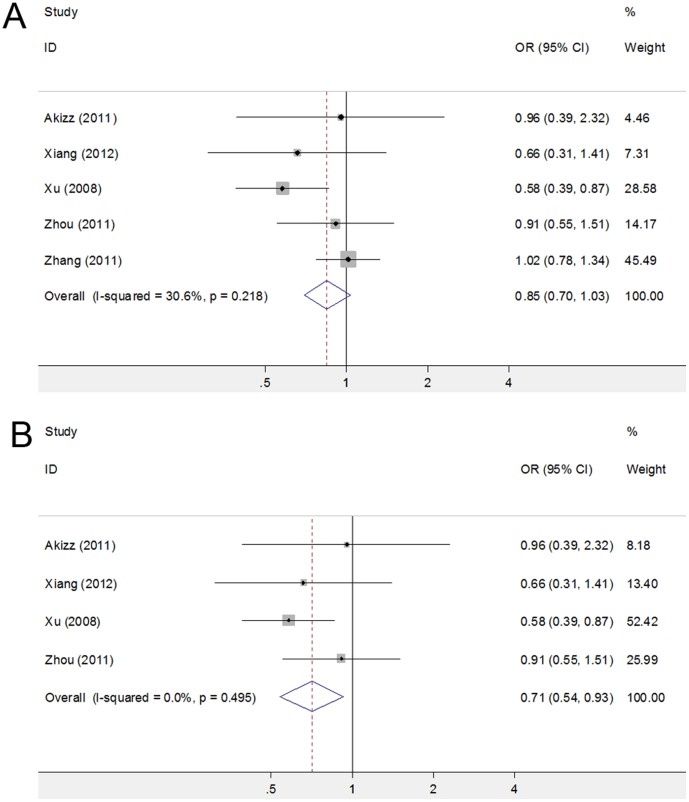
Forest plots before and after exclusion of Zhang et al’s study in the comparison between rs2910164 genotype CC and GG. A. Before exclusion of the study from Zhang et al. B. After exclusion of Zhang et al’s study. The squares and horizontal lines correspond to the study-specific OR and 95% CI. The area of the squares reflects the study specific weight. The diamond represents the pooled OR and 95% CI.

Every single study involved in this meta-analysis was deleted each time to examine the influence of the individual data set to the pooled ORs. For rs2910164, the study from Zhang *et al.* [17] showed significant effect on the pooled OR. After exclusion of this study in the genotype comparison of CC versus GG, the heterogeneity test remained negative, however, pooled OR changed from 0.850 (95% CI 0.701–1.031, *P* = 0.10) to 0.709 (95% CI 0.539–0.931, *P* = 0.01)(Figure 2), indicating the study from Zhang *et al.* may have a major influence on the estimation of the potential association. To explore possible factors that may influence the stability of this comparison, we conducted stratified analysis by HBV infection status, which is a well-known predisposing factor of HCC [4], in control populations. With HBV negative controls, the pooled OR was 0.957 (95% CI 0.767–1.195, *P* = 0.70), indicating no significant difference of susceptibility to HCC between CC and GG genotypes. Similarly, synthesis of the data with HBV positive control population obtained a pooled OR without statistical significance (OR = 0.855, 95% CI 0.550–1.330, P = 0.49). The consistency of the stratified analysis suggests that HBV infection status in control populations may not be the factor that affected the stability of the comparison between CC and GG genotypes. Caution should be made when interpreting the result of the comparison between CC and GG genotype.

## Discussion

The research on miRNAs has led to novel insights into the molecular mechanism of HCC [30]_ENREF_22. Since each miRNA may have hundreds of target genes, even a minor inherited variation in miRNAs could have profound impact on the expression of numerous genes including oncogenes and tumor suppressor genes, thus contributes to individual’s susceptibility to cancers [6]. Recently, much effort has been made towards elucidating the role of SNPs in miRNA-coding region and their effect on susceptibility to HCC. Current reports have revealed that SNPs in miRNA-coding region may involve in the process of hepatocarcinogenesis and contribute to individual susceptibility to the development of HCC. Better understanding of the relationship between these polymorphisms and HCC may help improve the management of this deadly disease by the prevention and early detection of HCC in high risk populations [3]. Two functional SNPs, miR-146a rs2910164 [10]–[13], [17] and miR-196a2 rs11614913 [14]–[17], are indicated to be associated with susceptibility to HCC. However, due to the limited sample size and the potential bias of case selection, the associations observed are controversial and inconclusive. In this meta-analysis, we systematically summarized 8 eligible case-control studies on the association between miR-146a rs2910164 or miR-196a2 rs11614913 polymorphisms and the risk of HCC.

miR-146a rs2910164 is located in the stem region opposite to the mature miR-146a sequence. This G>C polymorphism results in a change from G:U pair to C:U mismatch in the stem structure of miR-146a precursor. Several case-control studies have investigated the association between miR-146a rs2910164 polymorphism and risks of various cancers.[31]–[35] The C allele gene displayed decreased production of mature miR-146a compared with G allele and led to less efficient inhibition of target genes including IL-1 receptor-associated kinase 1 (IRAK1), TNF receptor-associated factor 6 (TRAF6) and papillary thyroid carcinoma 1 gene (PTC1) [36]. Xu et al. showed that the G allele represented elevation of mature miR-146a and subsequently increased cell proliferation and colony formation [12]. More importantly, Xu et al. first observed the association between SNP miR-146a rs2910164 and susceptibility to HCC. However, the results from other groups reported lack of association of miR-146a rs2910164 and the risk for HCC [10], [11], [17], or only associations between miR-146a SNP and early onset or better Child-Pugh grade in HCC cases [13].

In this meta-analysis, a total of 5 case-control studies were analyzed to provide a comprehensive assessment of the association between miR-146a rs2910164 polymorphism and HCC. Our results did not support a genetic association between rs2910164 and susceptibility to HCC. Neither allele frequency nor genotype distribution was significantly associated with susceptibility to HCC. Since the incidence of gene polymorphisms may vary between different ethnic groups and this variation may interfere with the detection of minor effect of SNPs on HCC risk, subgroup analysis in Chinese population was performed to further explore the potential association between rs2910164 and the risk of HCC. However, even within the same ethnic group, no association of statistical significance was observed. Sensitivity analysis showed that the study from Zhang *et al.* [17] had a significant influence on the pooled OR, after deleting the data set from this study, pooled OR for CC versus GG changed from 0.85 (95% CI 0.70–1.03, *P* = 0.10) to 0.71 (95% CI 0.54–0.93, *P* = 0.01). Zhang et al’s study with a relatively large sample size may be the source of potential heterogeneity or bias in the comparison between genotype CC and GG and could cause a major influence on the estimation of the association between rs2910164 polymorphism and HCC risk. Since HBV infection is a well-established risk factor for the development of HCC [4], HBV infection status in control population could be the source of bias and may explain the instability of the comparison between genotype CC and GG. However, stratified analysis by HBV status in controls showed consistent results suggesting no significant difference of susceptibility to HCC existed between CC and GG genotypes, which suggested HBV status may not be the main factor that influenced the stability of this comparison. However, lack of detailed information of both cases and controls prevented us from further exploring the potential source of the inconsistency observed in sensitivity analysis. In conclusion, possible association between rs2910164 polymorphism and HCC risk should not be ruled out and this result should be interpreted cautiously.

Another SNP shown to have potential relationship to the risk for HCC is rs11614913 in miR-196a2. It is reported that C allele of rs11614913 increased the expression of mature miR-196a2 in HCC tissues [15]. Of note, several genes involved in carcinogenesis including homeobox (HOX) and annexin A1 (ANXA1) have been reported to be the target of miR-196a2. HOX genes encode important transcription factors during normal development and in carcinogenesis [37]. The disorder of HOX proteins was suggested to play a crucial role in hepatocarcinogenesis and the progression of HCC [38]. Acting as a mediator of apoptosis and inhibitor of cell proliferation, ANXA1 participates in various physiological and pathological processes [39]–[42]. Masaki et al. demonstrated that ANXA1 participated in the malignant transformation of HCC and was closely related to the malignant behavior of HCC [43]. Therefore, alterations in miR-196a2 may contribute to susceptibility to HCC through the deregulation of target genes including HOX and ANXA1. Indeed, recent studies have reported the association of miR-196a2 rs11614913 polymorphism with HCC risk [14]–[16]. However, in another study, no association between rs11614913 and HCC was observed [17].

4 studies on the relationship between rs11614913 and susceptibility to HCC were included in our meta-analysis. To our surprise, we failed to find any association between rs11614913 polymorphism and the risk for HCC in any of the examined genetic models. Similarly, subgroup analysis in Chinese population also showed no association of miR-196a2 rs11614913 polymorphism with susceptibility to HCC. Sensitivity analysis found no significant influence of any single study on pooled ORs, indicating the stability of this meta-analysis was acceptable.

### Comparisons with other Meta-analysis

Several previous meta-analyses systematically reviewed the potential association of the two common polymorphisms rs2910164 and rs11614913 with susceptibility to cancer [18]–[25]. Two of them investigated rs2910164 in miR-146a [19], [22] while four studies focused on rs11614913 in miR-196a2 [18], [21], [23], [25]. Meta-analyses from Tian et al [24] and Xu et al [20] covered both of the two SNPs. These meta-analyses reported statistically significant association between the two common SNPs and susceptibility to cancer without pre-specified tissue origin. However, the major concern is clinical heterogeneity brought by inherent difference between cancers from distinct tissue origins, which may limit the reliability of the conclusions of the previous meta-analyses. Only two meta-analyses evaluated the role of rs2910164 and rs11614913 with pre-specified cancer type as breast cancer [44], [45]. With more studies included than previous meta-analysis by Gao et al, Lian et al reported association between CC homozygote of rs2910164 may contribute to breast cancer susceptibility in Europeans which was not detected in the meta-analysis by Gao et al, indicating insufficient data included may limit the power of meta-analysis. To date, there is no meta-analysis with focus on the association of rs2910164 and rs11614913 with HCC risk. Some of the previous meta-analyses explored the relationship between rs11614913 and HCC by means of subgroup analysis [18], [20], [21], [23]. Unfortunately, the results were controversial across the meta-analyses which may at least partly due to the fact that none of these meta-analyses included all available studies concerning the association between rs11614913 and susceptibility to HCC. With regard to rs2910164, none of the meta-analyses performed subgroup analysis in HCC and similarly, several association studies with eligible data were left out in their analyses. In the present meta-analysis, with focus on HCC, we performed comprehensive literature search in multiple databases without limiting publication language and date and included all available up-to-date evidence on the association of the two common SNPs and susceptibility to HCC. We conducted quantitative data synthesis in allele frequency, additive model, dominant model and recessive model and found none of the SNPs was associated with the risk of HCC in all of the genetic models tested. Our meta-analysis, though with limitations, concludes that there is no significant association of rs2910164 in miR-146a and rs11614913 in miR-196a2 with susceptibility to HCC with stronger evidence compared with previous meta-analyses.

### Limitations

To the best of our knowledge, this is the first meta-analysis evaluating the potential association between two common polymorphisms rs2910164 in miR-146a and rs11614913 in miR-196a2 and susceptibility to hepatocellular carcinoma. However, caution should be made when interpreting the results due to some limitations of this meta-analysis. Firstly, heterogeneity was detected in some comparisons of rs11614913 and sensitivity analysis showed that the study from Zhang *et al.* [17] had a significant influence on the evaluation of potential association between rs2910164 and the risk for HCC. Stratified analysis showed that HBV infection status in control population was not the cause of inconsistency of the result whereas insufficient information prevented us from further exploring the source of the instability. Importantly, it should be acknowledged that potential heterogeneity and bias may distort the results from this meta-analysis. Secondly, even after assembling the existed evidences, the sample size is still relatively small thus may not be sufficient enough to detect possible minor effects of miR-146a rs2910164 and miR-196a2 rs11614913 polymorphisms on susceptibility to HCC. Thirdly, lack of available data prevented an adjustment for subgroup factors including age, HBV/HCV infection status, alcohol consumption and gender, etc. These factors could influence the estimation of the slight associations between SNPs and susceptibility to HCC by interacting with genetic factors. Indeed, several studies report that the association of the two SNPs with HCC risk only existed in male population [12], [16]. At last, the included studies were carried out in Asians (Chinese population) and Caucasians (Turkish population). Absence of data from other ethnics makes a more comprehensive evaluation of the association between the two SNPs and susceptibility to HCC not possible.

In summary, despite the limitation, this meta-analysis suggests that miR-146a rs2910164 and miR-196a2 rs11614913 polymorphisms may not be associated with the risk of HCC. Well-designed studies with larger sample size and more ethnic groups should be considered to further clarify the association. Moreover, other factors such as gender, age, HBV/HCV infection status and alcohol consumption should also be considered in future studies.

## Supporting Information

Table S1 **MOOSE checklist.**
(DOC)
